# (Diethyl ether){1-[2-(1-methyl-1*H*-imidazol-2-yl-κ*N* ^3^)-1,1-diphenyl­ethyl]-(1,2,3,3a,7a-η)-inden­yl}lithium(I)

**DOI:** 10.1107/S1600536809011556

**Published:** 2009-04-02

**Authors:** Guofeng Sun, Chong Tian, Wanli Nie, Maxim V. Borzov

**Affiliations:** a The North-West University of Xi’an, College of Chemistry and Material Science, Taibai Bei avenue 229, Xi’an 710069, Shaanxi Province, People’s Republic of China

## Abstract

In the title compound, [Li(C_27_H_23_N_2_)(C_4_H_10_O)], the Li atom possesses a nearly planar trigonal coordination environment (assuming the cyclo­penta­dienyl ring of the indenyl group occupies one coordination place). The diethyl ether ligand adopts a nearly planar W-type conformation.

## Related literature

For the structural parameters of compounds with the (η^5^-1*H*-inden­yl)lithium fragment, see: Schumann *et al.* (2001 ▶); Cipot *et al.* (2003 ▶); Wang *et al.* (2005 ▶); Dinnebier *et al.* (1999 ▶); Feng *et al.* (2005 ▶); Faure *et al.* (2000 ▶); Cheng *et al.* (2004 ▶); Jones & Alan (2005 ▶). For the (η^5^-9*H*-fluoren­yl)lithium counterpart of a similar structure, see: Culp & Cowley (1996 ▶). For the synthesis, see: Krut’ko *et al.* (2006 ▶).
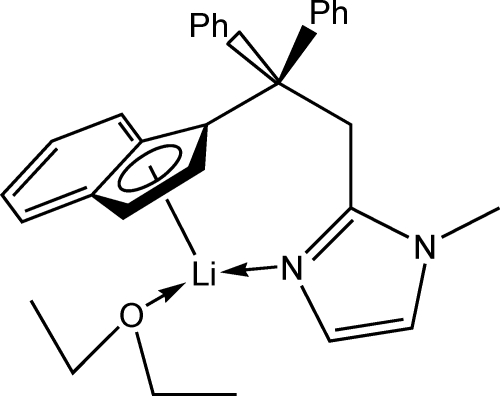


## Experimental

### 

#### Crystal data

[Li(C_27_H_23_N_2_)(C_4_H_10_O)]
*M* *_r_* = 456.53
Orthorhombic, 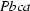

*a* = 19.620 (2) Å
*b* = 12.8763 (13) Å
*c* = 20.698 (2) Å
*V* = 5229.0 (9) Å^3^
*Z* = 8
Mo *K*α radiation
μ = 0.07 mm^−1^
*T* = 293 K
0.32 × 0.21 × 0.11 mm

#### Data collection

Bruker SMART APEXII CCD diffractometer
Absorption correction: multi-scan (*SADABS*; Sheldrick, 1996 ▶) *T*_min_ = 0.978, *T*_max_ = 0.992
24751 measured reflections
4595 independent reflections
2357 reflections with *I* > 2σ(*I*)
*R*_int_ = 0.056

#### Refinement

*R*[*F* ^2^ > 2σ(*F* ^2^)] = 0.048
*wR*(*F* ^2^) = 0.152
*S* = 1.02
4595 reflections
320 parameters
7 restraints
H-atom parameters constrained
Δρ_max_ = 0.32 e Å^−3^
Δρ_min_ = −0.20 e Å^−3^

### 

Data collection: *APEX2* (Bruker, 2007 ▶); cell refinement: *SAINT* (Bruker, 2007 ▶); data reduction: *SAINT*; program(s) used to solve structure: *SHELXS97* (Sheldrick, 2008 ▶); program(s) used to refine structure: *SHELXL97* (Sheldrick, 2008 ▶); molecular graphics: *SHELXTL* (Sheldrick, 2008 ▶); software used to prepare material for publication: *SHELXL97*.

## Supplementary Material

Crystal structure: contains datablocks I, global. DOI: 10.1107/S1600536809011556/dn2437sup1.cif

Structure factors: contains datablocks I. DOI: 10.1107/S1600536809011556/dn2437Isup2.hkl

Additional supplementary materials:  crystallographic information; 3D view; checkCIF report

## Figures and Tables

**Table:** 

Li1—N2	2.004 (5)
Li1—O1	2.015 (4)
Li—Cp	2.041 (4)

**Table:** 

N2—Li1—O1	105.98 (19)
C2—C3—N2	110.5 (2)

**Fig. 1.**
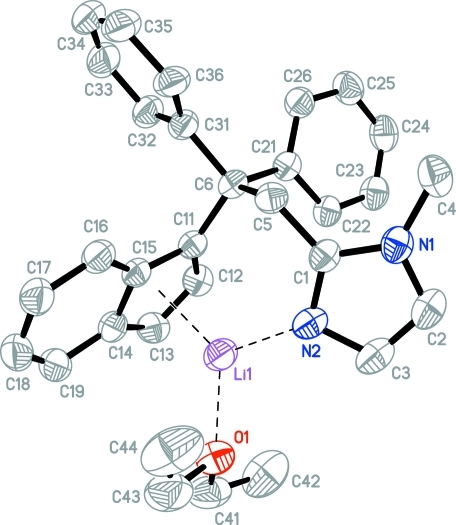
Molecular structure of 1. Thermal ellpsoids are drawn at 30% probability level. All hydrogen atoms are omitted for clarity. Bonds from Li1 to N2, O1, and Cpcent are drawn as dashed lines.

## supplementary crystallographic information

### Comment

Lithium indenides are important synthetic precursors of the related Group 4 transition metal complexes known as pre-catalysts for homogeneous a-olefin polymerization. Surprisingly, only few of them were structurally characterized (Schumann *et al.*, 2001; Cipot *et al.*, 2003; Wang *et al.*, 2005; Dinnebier *et al.*, 1999; Feng *et al.*, 2005; Faure *et al.*, 2000; Cheng *et al.*, 2004; Jones & Alan, 2005).

In the molecule of I, the Li-atom possesses a nearly planar trigonal coordination environment [assuming the Cp-ring of the indenyl group occupying one coordination place; sum of the valent angles N2—Li1—O1 (105.98 (19)°), N2—Li1—Cp_cent_ (122.3°) and O1—Li1—Cp_cent_ (131.7°) equals 360.0°] (Fig. 1). The solvent molecule adopts a nearly planar W-conformation [methyl group atoms C42 and C44 deviate from the (C41, O1, C43) plane by 0.436 (6) and -0.045 (6) Å, respectively] and is involved, as a rigid group, into a rocking motion around O–Li bond (the max. principal thermal ellipsoid axes for the ether molecule atoms are nearly tangent in respect to rotation around O1–Li1 bond). Li1 – r. m. s. plane (C11 through C15) distance is 2.041 (4) Å (the same as Li1–Cp_cent_ one). Coordination environment of O1 atom is essentially non-planar, with the angle between O1–Li1 bond and the normal to (C41, O1, C43) plane being equal to 54.1°.

### Experimental

All operations were performed in all-sealed glassware with application of the high-vacuum line technique (residual partial pressure of non-condensable gases below 1.5 × 10^-3^ torr), with traces of oxygen and moisture excluded. Solutions of (1-methyl-1*H*-imidazol-2-yl)methyllithium (adduct with THF 2: 1) [see (Krut'ko *et al.*, 2006)] (2.234 g, 16.2 mmol) and 1-diphenylmethylidene-1*H*-indene (4.535 g, 16.2 mmol) in THF (total amount 100 ml) were mixed and heated at 60 °C for 1 h. The dark-blue solution was concentrated till dryness and the rest was extracted with diethyl ether that gave 7.42 g (86%) of the lithium salt Ia (adduct with TWO molecules of THF, ^1^H NMR spectral data) as dark-blue crystalline material. Single crystal of I (adduct with one molecule of THF) suitable for X-ray diffraction analysis was grown up from hot ether solution (slow cooling within 60 - 30 °C range, sealed vessel). Green crystals of I in THF-*d*_8_ form pink solution.

^1^H NMR (THF-*d*_8_, 22 °C) δ = 3.03 (s, 3 H, NCH_3_), 4.03 (s, 2 H, CH_2_), 5.95, 6.46 (both d, 1 H and 1 H, ^3^*J* = 3.4 Hz, CH=CH in indenide), 6.69 (both d, 1 H and 1 H, ^3^*J* = 1.2 Hz, CH=CH in imidazole), 6.24, 6.39, 6.79, 7.30 (all m, all 1 H, benz-CH in indene), 6.98–7.18 (m, 10 H, Ph-ring protons). ^1^H NMR (THF-*d*_8_, 22 °C) δ = 32.35 (NCH_3_), 37.30 (CH_2_), 55.98 (CPh_2_), 91.62, 108.97 (CH=CH in indene), 113.83, 114.02, 117.36, 120.31, 120.47, 120.64, 125.64, 126.25, 127.25 (double int.), 129.78, 130.56, 131.78 (unambiguous interpretement is not possible), 149.06 (C in Ph), 150.99 (N—C=N).

### Refinement

Non-H atoms were refined anisotropically. H atoms were treated as riding atoms with distances C—H = 0.96 (CH_3_), 0.97 (CH_2_), 0.93 Å (C_Ar_H), and *U*_iso_(H) = 1.5 *U*_eq_(C), 1.2 *U*_eq_(C), and 1.2 *U*_eq_(C), respectively. For Et_2_O atoms C41 through C44 and O1, the components of the anisotropic displacements along 1–2 and 1–3 directions were restrained to be the same with a standard uncertainty of 0.005 Å^2^.

### Crystal data

**Table:**

[Li(C_27_H_23_N_2_)(C_4_H_10_O)]	*F*(000) = 1952
*M**_r_* = 456.53	*D*_x_ = 1.160 Mg m^−^^3^
Orthorhombic, *P**b**c**a*	Mo *K*α radiation, λ = 0.71073 Å
Hall symbol: -P 2ac 2ab	Cell parameters from 4629 reflections
*a* = 19.620 (2) Å	θ = 2.2–21.0°
*b* = 12.8763 (13) Å	µ = 0.07 mm^−^^1^
*c* = 20.698 (2) Å	*T* = 293 K
*V* = 5229.0 (9) Å^3^	Prism, green
*Z* = 8	0.32 × 0.21 × 0.11 mm

### Data collection

**Table:**

Bruker SMART APEX diffractometer	4595 independent reflections
Radiation source: fine-focus sealed tube	2357 reflections with *I* > 2σ(*I*)
graphite	*R*_int_ = 0.056
φ and ω scans	θ_max_ = 25.0°, θ_min_ = 2.0°
Absorption correction: multi-scan (*SADABS*; Sheldrick, 1996)	*h* = −20→23
*T*_min_ = 0.978, *T*_max_ = 0.992	*k* = −15→15
24751 measured reflections	*l* = −24→24

### Refinement

**Table:**

Refinement on *F*^2^	Secondary atom site location: difference Fourier map
Least-squares matrix: full	Hydrogen site location: inferred from neighbouring sites
*R*[*F*^2^ > 2σ(*F*^2^)] = 0.048	H-atom parameters constrained
*wR*(*F*^2^) = 0.152	*w* = 1/[σ^2^(*F*_o_^2^) + (0.0739*P*)^2^] where *P* = (*F*_o_^2^ + 2*F*_c_^2^)/3
*S* = 1.02	(Δ/σ)_max_ < 0.001
4595 reflections	Δρ_max_ = 0.32 e Å^−^^3^
320 parameters	Δρ_min_ = −0.20 e Å^−^^3^
7 restraints	Extinction correction: *SHELXL97* (Sheldrick, 2008), Fc^*^=kFc[1+0.001xFc^2^λ^3^/sin(2θ)]^-1/4^
Primary atom site location: structure-invariant direct methods	Extinction coefficient: 0.0022 (5)

### Special details

**Table:**

Geometry. All e.s.d.'s (except the e.s.d. in the dihedral angle between two l.s. planes) are estimated using the full covariance matrix. The cell e.s.d.'s are taken into account individually in the estimation of e.s.d.'s in distances, angles and torsion angles; correlations between e.s.d.'s in cell parameters are only used when they are defined by crystal symmetry. An approximate (isotropic) treatment of cell e.s.d.'s is used for estimating e.s.d.'s involving l.s. planes.
Refinement. Refinement of *F*^2^ against ALL reflections. The weighted *R*-factor *wR* and goodness of fit *S* are based on *F*^2^, conventional *R*-factors *R* are based on *F*, with *F* set to zero for negative *F*^2^. The threshold expression of *F*^2^ > σ(*F*^2^) is used only for calculating *R*-factors(gt) *etc*. and is not relevant to the choice of reflections for refinement. *R*-factors based on *F*^2^ are statistically about twice as large as those based on *F*, and *R*- factors based on ALL data will be even larger.

### Fractional atomic coordinates and isotropic or equivalent isotropic displacement parameters (Å^2^)

**Table:**

	*x*	*y*	*z*	*U*_iso_*/*U*_eq_	
Li1	0.13031 (19)	0.1533 (3)	0.39839 (18)	0.0771 (11)	
N1	0.07860 (10)	−0.12724 (15)	0.31605 (9)	0.0698 (6)	
N2	0.09933 (9)	0.00828 (15)	0.37892 (9)	0.0687 (5)	
C1	0.12154 (11)	−0.04750 (18)	0.32932 (10)	0.0599 (6)	
C2	0.02674 (13)	−0.1227 (2)	0.35940 (13)	0.0855 (8)	
H2	−0.0111	−0.1685	0.3622	0.103*	
C3	0.03967 (13)	−0.0401 (2)	0.39760 (13)	0.0844 (8)	
H3	0.0116	−0.0183	0.4325	0.101*	
C4	0.08482 (13)	−0.2031 (2)	0.26429 (13)	0.0920 (8)	
H4A	0.0831	−0.1675	0.2225	0.138*	
H4B	0.0472	−0.2530	0.2671	0.138*	
H4C	0.1283	−0.2399	0.2684	0.138*	
C5	0.18531 (10)	−0.02763 (18)	0.29257 (10)	0.0609 (6)	
H5A	0.1939	−0.0875	0.2637	0.073*	
H5B	0.2237	−0.0236	0.3235	0.073*	
C6	0.18485 (9)	0.07348 (17)	0.25096 (9)	0.0568 (6)	
C11	0.17977 (10)	0.16801 (17)	0.29535 (9)	0.0578 (6)	
C12	0.13052 (12)	0.24721 (19)	0.29777 (11)	0.0704 (6)	
H12	0.0918	0.2513	0.2703	0.084*	
C13	0.14622 (13)	0.31901 (19)	0.34603 (12)	0.0781 (7)	
H13	0.1199	0.3782	0.3573	0.094*	
C14	0.20755 (13)	0.2886 (2)	0.37489 (11)	0.0714 (7)	
C15	0.22949 (11)	0.19385 (19)	0.34335 (10)	0.0629 (6)	
C16	0.29167 (12)	0.1485 (2)	0.36330 (10)	0.0745 (7)	
H16	0.3073	0.0864	0.3434	0.089*	
C17	0.32949 (13)	0.1942 (3)	0.41150 (13)	0.0918 (9)	
H17	0.3714	0.1636	0.4243	0.110*	
C18	0.30745 (17)	0.2848 (3)	0.44184 (12)	0.0943 (9)	
H18	0.3344	0.3143	0.4753	0.113*	
C19	0.24817 (16)	0.3318 (2)	0.42462 (12)	0.0883 (8)	
H19	0.2340	0.3935	0.4459	0.106*	
C21	0.12573 (10)	0.06018 (17)	0.20274 (10)	0.0585 (6)	
C22	0.05774 (11)	0.06908 (17)	0.22160 (11)	0.0648 (6)	
H22	0.0473	0.0915	0.2642	0.078*	
C23	0.00511 (12)	0.0457 (2)	0.17914 (14)	0.0782 (7)	
H23	−0.0408	0.0522	0.1932	0.094*	
C24	0.01830 (14)	0.0137 (2)	0.11771 (14)	0.0860 (8)	
H24	−0.0180	−0.0008	0.0887	0.103*	
C25	0.08479 (14)	0.0028 (2)	0.09833 (12)	0.0858 (8)	
H25	0.0946	−0.0209	0.0559	0.103*	
C26	0.13787 (12)	0.02615 (19)	0.14016 (10)	0.0714 (7)	
H26	0.1835	0.0187	0.1256	0.086*	
C31	0.25269 (11)	0.0834 (2)	0.21395 (9)	0.0674 (7)	
C32	0.26254 (12)	0.1724 (2)	0.17666 (11)	0.0826 (8)	
H32	0.2280	0.2241	0.1751	0.099*	
C33	0.32247 (17)	0.1862 (3)	0.14166 (13)	0.1068 (11)	
H33	0.3284	0.2460	0.1154	0.128*	
C34	0.37300 (17)	0.1127 (4)	0.14541 (17)	0.1193 (14)	
H34	0.4143	0.1226	0.1222	0.143*	
C35	0.36456 (14)	0.0259 (3)	0.18195 (15)	0.1050 (11)	
H35	0.4001	−0.0241	0.1842	0.126*	
C36	0.30438 (11)	0.0097 (2)	0.21600 (11)	0.0798 (7)	
H36	0.2986	−0.0518	0.2407	0.096*	
O1	0.10490 (8)	0.17992 (15)	0.49122 (8)	0.0859 (5)	
C41	0.06321 (18)	0.2679 (2)	0.50301 (14)	0.1134 (10)	
H41A	0.0871	0.3319	0.4893	0.136*	
H41B	0.0530	0.2735	0.5497	0.136*	
C42	−0.00153 (17)	0.2555 (3)	0.46558 (18)	0.1471 (15)	
H42A	0.0086	0.2570	0.4192	0.221*	
H42C	−0.0327	0.3123	0.4763	0.221*	
H42B	−0.0228	0.1890	0.4767	0.221*	
C43	0.15656 (18)	0.1729 (3)	0.54066 (14)	0.1278 (12)	
H43B	0.1350	0.1640	0.5835	0.153*	
H43A	0.1841	0.2373	0.5415	0.153*	
C44	0.19980 (19)	0.0846 (4)	0.52646 (17)	0.1513 (16)	
H44A	0.2289	0.1009	0.4893	0.227*	
H44B	0.1714	0.0242	0.5163	0.227*	
H44C	0.2283	0.0691	0.5641	0.227*	

### Atomic displacement parameters (Å^2^)

**Table:**

	*U*^11^	*U*^22^	*U*^33^	*U*^12^	*U*^13^	*U*^23^
Li1	0.073 (3)	0.080 (3)	0.079 (2)	−0.002 (2)	0.0148 (19)	0.000 (2)
N1	0.0699 (13)	0.0588 (14)	0.0808 (13)	−0.0005 (11)	0.0005 (10)	0.0118 (11)
N2	0.0691 (12)	0.0674 (13)	0.0694 (11)	0.0065 (11)	0.0155 (10)	0.0098 (10)
C1	0.0592 (14)	0.0545 (15)	0.0658 (13)	0.0027 (12)	0.0029 (11)	0.0134 (11)
C2	0.0740 (18)	0.080 (2)	0.1020 (19)	−0.0060 (15)	0.0127 (16)	0.0216 (17)
C3	0.0792 (18)	0.084 (2)	0.0903 (18)	0.0069 (15)	0.0261 (14)	0.0216 (16)
C4	0.105 (2)	0.0691 (18)	0.101 (2)	−0.0129 (15)	−0.0075 (16)	−0.0036 (16)
C5	0.0561 (13)	0.0621 (15)	0.0644 (13)	0.0083 (11)	0.0015 (10)	0.0001 (11)
C6	0.0505 (12)	0.0647 (15)	0.0553 (11)	0.0039 (10)	0.0036 (10)	0.0076 (10)
C11	0.0573 (13)	0.0586 (15)	0.0576 (12)	−0.0002 (11)	0.0056 (10)	0.0064 (11)
C12	0.0753 (16)	0.0618 (16)	0.0741 (15)	0.0017 (13)	−0.0015 (12)	0.0108 (13)
C13	0.096 (2)	0.0555 (16)	0.0830 (16)	0.0026 (14)	0.0119 (15)	0.0007 (13)
C14	0.0816 (18)	0.0638 (17)	0.0689 (15)	−0.0199 (14)	0.0126 (13)	0.0063 (13)
C15	0.0610 (14)	0.0717 (17)	0.0559 (12)	−0.0118 (12)	0.0086 (11)	0.0047 (11)
C16	0.0617 (15)	0.098 (2)	0.0638 (13)	−0.0071 (13)	0.0041 (12)	0.0044 (13)
C17	0.0727 (17)	0.131 (3)	0.0716 (16)	−0.0215 (18)	−0.0024 (14)	0.0006 (17)
C18	0.098 (2)	0.113 (3)	0.0716 (17)	−0.040 (2)	0.0002 (16)	−0.0015 (18)
C19	0.108 (2)	0.081 (2)	0.0754 (17)	−0.0330 (18)	0.0137 (16)	−0.0046 (14)
C21	0.0571 (14)	0.0577 (15)	0.0606 (13)	0.0016 (10)	−0.0011 (10)	0.0089 (11)
C22	0.0566 (14)	0.0634 (16)	0.0743 (14)	0.0019 (11)	−0.0034 (12)	0.0054 (12)
C23	0.0599 (15)	0.0754 (19)	0.0991 (19)	0.0021 (13)	−0.0080 (14)	0.0075 (15)
C24	0.0766 (19)	0.089 (2)	0.0928 (19)	−0.0124 (15)	−0.0246 (15)	0.0046 (16)
C25	0.097 (2)	0.092 (2)	0.0691 (16)	−0.0149 (16)	−0.0119 (14)	−0.0037 (14)
C26	0.0684 (16)	0.0810 (18)	0.0648 (14)	−0.0056 (13)	−0.0023 (12)	0.0010 (12)
C31	0.0559 (14)	0.0929 (19)	0.0535 (12)	−0.0075 (13)	0.0004 (11)	−0.0026 (13)
C32	0.0733 (17)	0.107 (2)	0.0674 (14)	−0.0184 (15)	0.0080 (12)	0.0037 (15)
C33	0.102 (2)	0.145 (3)	0.0737 (17)	−0.044 (2)	0.0214 (17)	−0.0050 (18)
C34	0.075 (2)	0.185 (4)	0.099 (2)	−0.038 (2)	0.0293 (19)	−0.045 (3)
C35	0.0653 (19)	0.158 (3)	0.092 (2)	0.0040 (19)	0.0110 (16)	−0.038 (2)
C36	0.0557 (15)	0.115 (2)	0.0692 (15)	0.0071 (15)	0.0063 (12)	−0.0151 (14)
O1	0.0886 (12)	0.0925 (14)	0.0765 (11)	0.0028 (10)	0.0090 (9)	0.0092 (9)
C41	0.157 (3)	0.084 (2)	0.100 (2)	0.008 (2)	0.0438 (19)	−0.0075 (17)
C42	0.115 (3)	0.135 (3)	0.191 (4)	0.033 (2)	0.034 (2)	0.039 (3)
C43	0.129 (3)	0.180 (4)	0.0742 (18)	−0.011 (2)	0.0022 (17)	0.020 (2)
C44	0.125 (3)	0.208 (4)	0.121 (3)	0.041 (3)	0.012 (2)	0.070 (3)

### Geometric parameters (Å, °)

**Table:**

Li1—N2	2.004 (5)	C18—C19	1.359 (4)
Li1—O1	2.015 (4)	C18—H18	0.9500
Li1—C15	2.315 (4)	C19—H19	0.9500
Li1—C11	2.351 (4)	C21—C26	1.388 (3)
Li1—C14	2.360 (5)	C21—C22	1.395 (3)
Li1—C12	2.408 (5)	C22—C23	1.389 (3)
Li1—C13	2.414 (5)	C22—H22	0.9500
Li—Cp	2.041 (4)	C23—C24	1.361 (3)
N1—C1	1.356 (3)	C23—H23	0.9500
N1—C2	1.358 (3)	C24—C25	1.372 (3)
N1—C4	1.455 (3)	C24—H24	0.9500
N2—C1	1.326 (3)	C25—C26	1.387 (3)
N2—C3	1.381 (3)	C25—H25	0.9500
C1—C5	1.486 (3)	C26—H26	0.9500
C2—C3	1.349 (4)	C31—C36	1.390 (3)
C2—H2	0.9500	C31—C32	1.395 (3)
C3—H3	0.9500	C32—C33	1.393 (3)
C4—H4A	0.9800	C32—H32	0.9500
C4—H4B	0.9800	C33—C34	1.373 (5)
C4—H4C	0.9800	C33—H33	0.9500
C5—C6	1.561 (3)	C34—C35	1.359 (5)
C5—H5A	0.9900	C34—H34	0.9500
C5—H5B	0.9900	C35—C36	1.391 (4)
C6—C11	1.528 (3)	C35—H35	0.9500
C6—C21	1.540 (3)	C36—H36	0.9500
C6—C31	1.541 (3)	O1—C41	1.419 (3)
C11—C12	1.406 (3)	O1—C43	1.443 (3)
C11—C15	1.431 (3)	C41—C42	1.496 (4)
C12—C13	1.396 (3)	C41—H41A	0.9900
C12—H12	0.9500	C41—H41B	0.9900
C13—C14	1.399 (3)	C42—H42A	0.9800
C13—H13	0.9500	C42—H42C	0.9800
C14—C19	1.416 (3)	C42—H42B	0.9800
C14—C15	1.449 (3)	C43—C44	1.448 (5)
C15—C16	1.414 (3)	C43—H43B	0.9900
C16—C17	1.376 (3)	C43—H43A	0.9900
C16—H16	0.9500	C44—H44A	0.9800
C17—C18	1.393 (4)	C44—H44B	0.9800
C17—H17	0.9500	C44—H44C	0.9800
			
N2—Li1—O1	105.98 (19)	C11—C15—C14	107.8 (2)
N2—Li1—C15	111.48 (19)	C16—C15—Li1	119.22 (18)
O1—Li1—C15	129.7 (2)	C11—C15—Li1	73.52 (15)
N2—Li1—C11	91.03 (16)	C14—C15—Li1	73.66 (16)
O1—Li1—C11	162.5 (2)	C17—C16—C15	120.0 (3)
C15—Li1—C11	35.73 (9)	C17—C16—H16	120.0
N2—Li1—C14	147.2 (2)	C15—C16—H16	120.0
O1—Li1—C14	103.28 (19)	C16—C17—C18	121.2 (3)
C15—Li1—C14	36.10 (10)	C16—C17—H17	119.4
C11—Li1—C14	59.22 (12)	C18—C17—H17	119.4
N2—Li1—C12	107.12 (19)	C19—C18—C17	121.4 (3)
O1—Li1—C12	137.7 (2)	C19—C18—H18	119.3
C15—Li1—C12	57.28 (12)	C17—C18—H18	119.3
C11—Li1—C12	34.33 (9)	C18—C19—C14	119.8 (3)
C14—Li1—C12	56.62 (12)	C18—C19—H19	120.1
N2—Li1—C13	140.6 (2)	C14—C19—H19	120.1
O1—Li1—C13	108.04 (19)	C26—C21—C22	116.83 (19)
C15—Li1—C13	58.04 (13)	C26—C21—C6	120.71 (18)
C11—Li1—C13	57.85 (12)	C22—C21—C6	122.00 (18)
C14—Li1—C13	34.06 (10)	C23—C22—C21	121.1 (2)
C12—Li1—C13	33.64 (10)	C23—C22—H22	119.5
C1—N1—C2	107.4 (2)	C21—C22—H22	119.5
C1—N1—C4	127.3 (2)	C24—C23—C22	121.0 (2)
C2—N1—C4	125.3 (2)	C24—C23—H23	119.5
C1—N2—C3	104.5 (2)	C22—C23—H23	119.5
C1—N2—Li1	124.08 (18)	C23—C24—C25	119.0 (2)
C3—N2—Li1	128.4 (2)	C23—C24—H24	120.5
N2—C1—N1	111.26 (19)	C25—C24—H24	120.5
N2—C1—C5	125.4 (2)	C24—C25—C26	120.6 (2)
N1—C1—C5	123.3 (2)	C24—C25—H25	119.7
C3—C2—N1	106.3 (2)	C26—C25—H25	119.7
C3—C2—H2	126.8	C25—C26—C21	121.5 (2)
N1—C2—H2	126.8	C25—C26—H26	119.3
C2—C3—N2	110.5 (2)	C21—C26—H26	119.3
C2—C3—H3	124.7	C36—C31—C32	118.5 (2)
N2—C3—H3	124.7	C36—C31—C6	123.9 (2)
N1—C4—H4A	109.5	C32—C31—C6	117.6 (2)
N1—C4—H4B	109.5	C33—C32—C31	120.6 (3)
H4A—C4—H4B	109.5	C33—C32—H32	119.7
N1—C4—H4C	109.5	C31—C32—H32	119.7
H4A—C4—H4C	109.5	C34—C33—C32	119.5 (3)
H4B—C4—H4C	109.5	C34—C33—H33	120.3
C1—C5—C6	114.90 (16)	C32—C33—H33	120.3
C1—C5—H5A	108.5	C35—C34—C33	120.7 (3)
C6—C5—H5A	108.5	C35—C34—H34	119.7
C1—C5—H5B	108.5	C33—C34—H34	119.7
C6—C5—H5B	108.5	C34—C35—C36	120.6 (3)
H5A—C5—H5B	107.5	C34—C35—H35	119.7
C11—C6—C21	115.41 (16)	C36—C35—H35	119.7
C11—C6—C31	106.80 (17)	C31—C36—C35	120.1 (3)
C21—C6—C31	109.73 (16)	C31—C36—H36	119.9
C11—C6—C5	109.45 (16)	C35—C36—H36	119.9
C21—C6—C5	105.62 (16)	C41—O1—C43	109.5 (3)
C31—C6—C5	109.79 (17)	C41—O1—Li1	116.3 (2)
C12—C11—C15	106.0 (2)	C43—O1—Li1	119.5 (2)
C12—C11—C6	130.09 (19)	O1—C41—C42	108.4 (3)
C15—C11—C6	123.91 (19)	O1—C41—H41A	110.0
C12—C11—Li1	75.08 (16)	C42—C41—H41A	110.0
C15—C11—Li1	70.76 (14)	O1—C41—H41B	110.0
C6—C11—Li1	120.53 (17)	C42—C41—H41B	110.0
C13—C12—C11	110.8 (2)	H41A—C41—H41B	108.4
C13—C12—Li1	73.38 (17)	C41—C42—H42A	109.5
C11—C12—Li1	70.59 (15)	C41—C42—H42C	109.5
C13—C12—H12	124.6	H42A—C42—H42C	109.5
C11—C12—H12	124.6	C41—C42—H42B	109.5
Li1—C12—H12	123.0	H42A—C42—H42B	109.5
C12—C13—C14	108.1 (2)	H42C—C42—H42B	109.5
C12—C13—Li1	72.97 (17)	O1—C43—C44	108.5 (3)
C14—C13—Li1	70.87 (16)	O1—C43—H43B	110.0
C12—C13—H13	126.0	C44—C43—H43B	110.0
C14—C13—H13	126.0	O1—C43—H43A	110.0
Li1—C13—H13	121.9	C44—C43—H43A	110.0
C13—C14—C19	133.2 (3)	H43B—C43—H43A	108.4
C13—C14—C15	107.4 (2)	C43—C44—H44A	109.5
C19—C14—C15	119.4 (3)	C43—C44—H44B	109.5
C13—C14—Li1	75.07 (17)	H44A—C44—H44B	109.5
C19—C14—Li1	120.11 (18)	C43—C44—H44C	109.5
C15—C14—Li1	70.24 (15)	H44A—C44—H44C	109.5
C16—C15—C11	134.0 (2)	H44B—C44—H44C	109.5
C16—C15—C14	118.2 (2)		
			
O1—Li1—N2—C1	−162.01 (19)	C15—Li1—C14—C19	−113.3 (3)
C15—Li1—N2—C1	−16.2 (3)	C11—Li1—C14—C19	−151.4 (3)
C11—Li1—N2—C1	13.9 (3)	C12—Li1—C14—C19	168.0 (3)
C14—Li1—N2—C1	−9.5 (5)	C13—Li1—C14—C19	131.6 (3)
C12—Li1—N2—C1	44.7 (3)	N2—Li1—C14—C15	−10.6 (4)
C13—Li1—N2—C1	49.1 (4)	O1—Li1—C14—C15	142.3 (2)
O1—Li1—N2—C3	40.8 (3)	C11—Li1—C14—C15	−38.10 (13)
C15—Li1—N2—C3	−173.4 (2)	C12—Li1—C14—C15	−78.75 (15)
C11—Li1—N2—C3	−143.4 (2)	C13—Li1—C14—C15	−115.1 (2)
C14—Li1—N2—C3	−166.7 (3)	C12—C11—C15—C16	−177.6 (2)
C12—Li1—N2—C3	−112.6 (2)	C6—C11—C15—C16	0.5 (4)
C13—Li1—N2—C3	−108.1 (3)	Li1—C11—C15—C16	115.0 (3)
C3—N2—C1—N1	0.5 (2)	C12—C11—C15—C14	1.2 (2)
Li1—N2—C1—N1	−161.28 (19)	C6—C11—C15—C14	179.34 (17)
C3—N2—C1—C5	−179.0 (2)	Li1—C11—C15—C14	−66.22 (17)
Li1—N2—C1—C5	19.3 (3)	C12—C11—C15—Li1	67.45 (18)
C2—N1—C1—N2	−0.4 (2)	C6—C11—C15—Li1	−114.4 (2)
C4—N1—C1—N2	178.2 (2)	C13—C14—C15—C16	178.70 (19)
C2—N1—C1—C5	179.1 (2)	C19—C14—C15—C16	−0.7 (3)
C4—N1—C1—C5	−2.3 (3)	Li1—C14—C15—C16	−114.8 (2)
C1—N1—C2—C3	0.1 (3)	C13—C14—C15—C11	−0.3 (2)
C4—N1—C2—C3	−178.5 (2)	C19—C14—C15—C11	−179.71 (18)
N1—C2—C3—N2	0.1 (3)	Li1—C14—C15—C11	66.12 (17)
C1—N2—C3—C2	−0.4 (3)	C13—C14—C15—Li1	−66.46 (19)
Li1—N2—C3—C2	160.3 (2)	C19—C14—C15—Li1	114.2 (2)
N2—C1—C5—C6	−68.5 (3)	N2—Li1—C15—C16	−72.6 (3)
N1—C1—C5—C6	112.1 (2)	O1—Li1—C15—C16	62.8 (4)
C1—C5—C6—C11	64.7 (2)	C11—Li1—C15—C16	−131.6 (3)
C1—C5—C6—C21	−60.1 (2)	C14—Li1—C15—C16	113.6 (3)
C1—C5—C6—C31	−178.38 (18)	C12—Li1—C15—C16	−169.6 (2)
C21—C6—C11—C12	−2.4 (3)	C13—Li1—C15—C16	150.3 (2)
C31—C6—C11—C12	119.9 (2)	N2—Li1—C15—C11	59.1 (2)
C5—C6—C11—C12	−121.3 (2)	O1—Li1—C15—C11	−165.6 (3)
C21—C6—C11—C15	179.99 (18)	C14—Li1—C15—C11	−114.8 (2)
C31—C6—C11—C15	−57.7 (2)	C12—Li1—C15—C11	−38.02 (13)
C5—C6—C11—C15	61.1 (2)	C13—Li1—C15—C11	−78.07 (15)
C21—C6—C11—Li1	93.7 (2)	N2—Li1—C15—C14	173.8 (2)
C31—C6—C11—Li1	−144.04 (18)	O1—Li1—C15—C14	−50.8 (3)
C5—C6—C11—Li1	−25.2 (2)	C11—Li1—C15—C14	114.8 (2)
N2—Li1—C11—C12	119.73 (19)	C12—Li1—C15—C14	76.77 (16)
O1—Li1—C11—C12	−73.6 (8)	C13—Li1—C15—C14	36.72 (13)
C15—Li1—C11—C12	−113.2 (2)	C11—C15—C16—C17	178.8 (2)
C14—Li1—C11—C12	−74.73 (16)	C14—C15—C16—C17	0.1 (3)
C13—Li1—C11—C12	−34.59 (14)	Li1—C15—C16—C17	−86.1 (3)
N2—Li1—C11—C15	−127.0 (2)	C15—C16—C17—C18	0.6 (4)
O1—Li1—C11—C15	39.6 (7)	C16—C17—C18—C19	−0.6 (4)
C14—Li1—C11—C15	38.51 (14)	C17—C18—C19—C14	0.0 (4)
C12—Li1—C11—C15	113.2 (2)	C13—C14—C19—C18	−178.5 (2)
C13—Li1—C11—C15	78.65 (15)	C15—C14—C19—C18	0.6 (3)
N2—Li1—C11—C6	−8.3 (2)	Li1—C14—C19—C18	83.6 (3)
O1—Li1—C11—C6	158.3 (7)	C11—C6—C21—C26	141.1 (2)
C15—Li1—C11—C6	118.7 (2)	C31—C6—C21—C26	20.4 (3)
C14—Li1—C11—C6	157.20 (19)	C5—C6—C21—C26	−97.8 (2)
C12—Li1—C11—C6	−128.1 (2)	C11—C6—C21—C22	−46.9 (3)
C13—Li1—C11—C6	−162.66 (19)	C31—C6—C21—C22	−167.6 (2)
C15—C11—C12—C13	−1.7 (2)	C5—C6—C21—C22	74.1 (2)
C6—C11—C12—C13	−179.66 (19)	C26—C21—C22—C23	−0.6 (3)
Li1—C11—C12—C13	62.8 (2)	C6—C21—C22—C23	−172.9 (2)
C15—C11—C12—Li1	−64.47 (17)	C21—C22—C23—C24	−0.2 (4)
C6—C11—C12—Li1	117.6 (2)	C22—C23—C24—C25	1.2 (4)
N2—Li1—C12—C13	174.9 (2)	C23—C24—C25—C26	−1.4 (4)
O1—Li1—C12—C13	34.8 (3)	C24—C25—C26—C21	0.6 (4)
C15—Li1—C12—C13	−80.20 (17)	C22—C21—C26—C25	0.4 (3)
C11—Li1—C12—C13	−119.8 (2)	C6—C21—C26—C25	172.8 (2)
C14—Li1—C12—C13	−36.81 (14)	C11—C6—C31—C36	121.2 (2)
N2—Li1—C12—C11	−65.30 (19)	C21—C6—C31—C36	−113.0 (2)
O1—Li1—C12—C11	154.6 (3)	C5—C6—C31—C36	2.7 (3)
C15—Li1—C12—C11	39.62 (13)	C11—C6—C31—C32	−58.3 (2)
C14—Li1—C12—C11	83.01 (16)	C21—C6—C31—C32	67.5 (2)
C13—Li1—C12—C11	119.8 (2)	C5—C6—C31—C32	−176.84 (19)
C11—C12—C13—C14	1.5 (3)	C36—C31—C32—C33	0.8 (3)
Li1—C12—C13—C14	62.59 (19)	C6—C31—C32—C33	−179.6 (2)
C11—C12—C13—Li1	−61.06 (18)	C31—C32—C33—C34	−1.8 (4)
N2—Li1—C13—C12	−7.7 (3)	C32—C33—C34—C35	1.2 (5)
O1—Li1—C13—C12	−156.2 (2)	C33—C34—C35—C36	0.3 (5)
C15—Li1—C13—C12	77.74 (16)	C32—C31—C36—C35	0.7 (3)
C11—Li1—C13—C12	35.30 (13)	C6—C31—C36—C35	−178.8 (2)
C14—Li1—C13—C12	116.7 (2)	C34—C35—C36—C31	−1.3 (4)
N2—Li1—C13—C14	−124.4 (3)	N2—Li1—O1—C41	−124.0 (2)
O1—Li1—C13—C14	87.1 (2)	C15—Li1—O1—C41	98.9 (3)
C15—Li1—C13—C14	−38.97 (14)	C11—Li1—O1—C41	69.9 (8)
C11—Li1—C13—C14	−81.41 (16)	C14—Li1—O1—C41	70.9 (3)
C12—Li1—C13—C14	−116.7 (2)	C12—Li1—O1—C41	16.4 (4)
C12—C13—C14—C19	178.5 (2)	C13—Li1—O1—C41	35.8 (3)
Li1—C13—C14—C19	−117.5 (3)	N2—Li1—O1—C43	101.1 (3)
C12—C13—C14—C15	−0.7 (2)	C15—Li1—O1—C43	−36.0 (4)
Li1—C13—C14—C15	63.25 (17)	C11—Li1—O1—C43	−65.0 (8)
C12—C13—C14—Li1	−63.95 (19)	C14—Li1—O1—C43	−64.0 (3)
N2—Li1—C14—C13	104.5 (4)	C12—Li1—O1—C43	−118.5 (3)
O1—Li1—C14—C13	−102.6 (2)	C13—Li1—O1—C43	−99.1 (3)
C15—Li1—C14—C13	115.1 (2)	C43—O1—C41—C42	−162.1 (3)
C11—Li1—C14—C13	77.00 (16)	Li1—O1—C41—C42	58.7 (3)
C12—Li1—C14—C13	36.35 (14)	C41—O1—C43—C44	−178.1 (3)
N2—Li1—C14—C19	−123.9 (4)	Li1—O1—C43—C44	−40.5 (4)
O1—Li1—C14—C19	29.0 (3)		
